# P048: Effect of methicillin-resistant Staphylococcus aureus bundle approach in a surgical intensive care unit

**DOI:** 10.1186/2047-2994-2-S1-P48

**Published:** 2013-06-20

**Authors:** EJ Kim, HS Oh, Jeong Suk Song, Young Rock Oh

**Affiliations:** 1 Infection control office, Seoul National University Hospital, Seoul, Korea, Republic Of; 2 Colleage of Nursing, Woosung Universty, Daejeon, Korea, Republic Of

## Introduction

Methicillin-resistant *Staphylococcus aureus* (MRSA) accounts for more than 70% of *S.aureus* isolates from tertiary hospital in Korea. Especially, MRSA is the major pathogen of nosocomial infections in intensive care units(ICUs).The MRSA bundle approach has been reported more effective than a single intervention to reduce MRSA in several studies.

## Objectives

The purpose of this study is to evaluate the effect of the MRSA bundle approach in reducing MRSA infections or colonizations in a surgical ICU of a university hospital in Korea.

## Methods

This study was conducted in surgical ICU(24 beds) in a university hospital for 8 months. The MRSA bundle approach for this study consisted of an active surveillance culture, hand hygiene, contact precaution, and decontamination of the environment and equipment. The MRSA incidence rate and MRSA nosocomial infection rate during the pre-intervention period(4 months) and those of the intervention period(4 months) were compared to identify the effect of the MRSA bundle approach. Data were analyzed by the Chi-square test, t-test and Mann-Whitney U test using the statistical software program SPSS(ver. 12.0). Statistical significance was accepted at the level of p<.05.

## Results

MRSA was newly isolated from clinical specimens in 31 patients (9.8%) during the pre-intervention period: therefore, the incidence rate of MRSA was 11.9 cases per 1,000 patient-days during the pre-intervention period. And MRSA was newly isolated from clinical specimens in 21 patients (5.4%) during the intervention period : thus, the incidence rate of MRSA was 7.6 cases per 1,000 patient-days during the intervention period(p=0.040).

MRSA nosocomial infections developed in 21 patients (6.8%) during the pre-intervention period: therefore, the nosocomial infection rate of MRSA was 8.4 cases per 1,000 patient-days. And MRSA nosocomial infections developed in 10 patients (2.6%) during the intervention period : thus, the nosocomial infection rate of MRSA was 3.6 cases per 1,000 patient-days(p=0.009).

## Conclusion

The MRSA bundle approach in the SICU effectively reduced the incidence rate and the nosocomial infection rate of MRSA.

## Disclosure of interest

None declared

